# Gb3‐cSrc complex in glycosphingolipid‐enriched microdomains contributes to the expression of p53 mutant protein and cancer drug resistance via β‐catenin–activated RNA methylation

**DOI:** 10.1096/fba.2020-00044

**Published:** 2020-09-02

**Authors:** Kartik R. Roy, Mohammad B. Uddin, Sagor C. Roy, Ronald A. Hill, John Marshall, Yu‐Teh Li, Jean Christopher Chamcheu, Hua Lu, Yong‐Yu Liu

**Affiliations:** ^1^ School of Basic Pharmaceutical and Toxicological Sciences College of Pharmacy University of Louisiana at Monroe Monroe Louisiana USA; ^2^ Department of Rare Genetic Disease Research Sanofi‐Genzyme R&D Center Genzyme, Framingham Massachusetts USA; ^3^ Department of Biochemistry and Molecular Biology Tulane University School of Medicine New Orleans Louisiana USA

**Keywords:** colon cancer, cSrc kinase, drug resistance, glycosphingolipid‐enriched microdomains, glycosphingolipids, missense mutation, p53 tumor suppressor

## Abstract

Glucosylceramide synthase (GCS) is a key enzyme catalyzing ceramide glycosylation to generate glucosylceramide (GlcCer), which in turn serves as the precursor for cells to produce glycosphingolipids (GSLs). In cell membranes, GSLs serve as essential components of GSL‐enriched microdomains (GEMs) and mediate membrane functions and cell behaviors. Previous studies showed that ceramide glycosylation correlates with upregulated expression of p53 hotspot mutant R273H and cancer drug resistance. Yet, the underlying mechanisms remain elusive. We report herewith that globotriaosylceramide (Gb3) is associated with cSrc kinase in GEMs and plays a crucial role in modulating expression of p53 R273H mutant and drug resistance. Colon cancer cell lines, either WiDr homozygous for missense‐mutated *TP53* (R273H^+/+^) or SW48/TP53‐Dox bearing heterozygous *TP53* mutant (R273H^/+^), display drug resistance with increased ceramide glycosylation. Inhibition of GCS with Genz‐161 (GENZ 667161) resensitized cells to apoptosis in these p53 mutant‐carrying cancer cells. Genz‐161 effectively inhibited GCS activity, and substantially suppressed the elevated Gb3 levels seen in GEMs of p53‐mutant cells exposed to doxorubicin. Complex formation between Gb3 and cSrc in GEMs to activate β‐catenin was detected in both cultured cells and xenograft tumors. Suppression of ceramide glycosylation significantly decreased Gb3‐cSrc in GEMs, β‐catenin, and methyltransferase‐like 3 for m^6^A RNA methylation, thus altering pre‐mRNA splicing, resulting in upregulated expression of wild‐type p53 protein, but not mutants, in cells carrying p53 R273H. Altogether, increased Gb3‐cSrc complex in GEMs of membranes in response to anticancer drug induced cell stress promotes expression of p53 mutant proteins and accordant cancer drug resistance.

## 1 INTRODUCTION

Ceramide glycosylation generates glycosphingolipids (GSLs) and modulates cell processes in response to environmental stress, including assault by various anticancer drugs. Glucosylceramide synthase (GCS) is a rate‐limiting enzyme in GSL synthesis, catalyzing ceramide (Cer) glycosylation that converts Cer into glucosylceramide (GlcCer) at the *cis*‐membrane of Golgi apparatus, thereby providing GlcCer as a precursor for various GlcCer‐based GSLs.^1^, ^2^ GlcCer is further converted to lactosylceramide (LacCer), which is the biosynthetic branching point for the formation of major GSLs, including globo‐series (globotriaosylceramide Gb3, globopentaosylceramide Gb5, etc.) and ganglio‐series (monosialogangliosides GM2, GM3; disialogangliosides GD2, GD3, etc.).^3^, ^4^ GSLs and other components, including sphingolipids, sterols (in particular cholesterol) and membrane‐associated proteins, alter the basic lipid bilayer to produce “lipid rafts” in eukaryotic membrane. These lipid rafts serve as transient, relatively ordered membrane platforms for many different molecules that are important in crucial intracellular signaling pathways.^5^, ^6^, ^7^, ^8^ A GSL‐enriched microdomain (GEM) is a noncaveolar lipid raft, rather than a caveolar one (marked by the presence of caveolins (Cav‐1, −2 and −3)), it is unequal in enrichment of GSLs, and marked with prominent flotillins (Flot‐1/reggie‐2 and Flot‐2/reggie‐1).^9^, ^10^, ^11^, ^12^ GSLs are tightly associated with membrane proteins and signal transducers in GEMs, and facilitate the downstream functional effects of these proteins, such as Src family kinases.^13^, ^14^, ^15^ Previous reports indicate that certain globo‐series GSLs, in particular Gb3, play important roles in modulating transcription of gene expression via cSrc and β‐catenin signaling pathways.^16^, ^17^ The active proto‐oncogene tyrosine‐protein kinase cSrc elevates the levels of transcriptional activator β‐catenin, in turn upregulating the expression of cyclin D1, c‐Myc, multidrug resistance 1 (MDR1), fibroblast growth factor 2 (FGF2), Her2/Her3, and p53 mutant proteins.^16^, ^17^, ^18^, ^19^, ^20^ Under drug treatments, increased Cer glycosylation and cellular GSLs may modulate GEMs so as to promote expression of particular proteins, including MDR1, FGF2, and even p53 mutants protecting cells, including cancer cells, against anticancer drugs.^16^, ^17^, ^20^

Missense p53 mutant proteins not only lack the tumor suppressor activity of wild‐type p53 but also exhibit oncogenic gain‐of‐function (GOF).^21^, ^22^, ^23^ The p53 protein, encoded by human gene *TP53*, functions as an essential tumor suppressor that stabilizes the genome with respect to the propensity for neoplastic transformation in normal cells or tissues. Acting as a nuclear homotetrameric transcription factor, p53 promotes the expression of its responsive target genes, including p21, Bax, Puma, and others, whereby p53‐dependent cell proliferation arrest or apoptosis is executed in response to genotoxic stress.^21^ It has been reported that *TP53* is mutated in approximately 42% of cancer cases, with occurrence in almost all types of cancers. Among these mutations, about 75% are missense mutations that can encode full‐length mutant proteins.^24^ However, p53 mutants are observed in more than 80% of metastatic cancers or recurred cancers, such as those of ovaries and colon.^25^, ^26^ Missense mutations at codons 175, 248, and 273 constitute approximately 19% of all p53 genetic alterations, thus these codons are referred to as mutation hotspots, DNA base substitutions at which are prevalently seen in cancers of ovaries, pancreas, colon, and lungs^24^ (http://p53.free.fr/Database/p53_cancer/all_cancer.html). In addition to other oncogenic effects on tumor progression, p53 missense mutants are causative of cancer drug resistance.^20^, ^27^, ^28^ Restoring the expression of wild‐type p53 or reactivating p53 function resensitizes cancer cells carrying *TP53* mutations to anticancer treatments.^22^, ^29^, ^30^, ^31^

DNA damage and cell stress upon treatments with anticancer drugs, such as doxorubicin, often cause increased ceramide glycosylation^32^, ^33^ and upregulated expression of the *TP53* gene, including accumulation of mutants.^22^, ^34^ To understand how cancer cells carrying gene mutations respond to anticancer drugs to gain resistance, we examined Cer glycosylation and GEMs toward identifying their roles in regulating mutant protein expression and cell survival.

## 2 MATERIALS AND METHODS

### 2.1 Cell lines and culture

Cells of the human colon cancer SW48 line, and of its corresponding SW48/TP53 missense mutant (p53 R273H^/^^+^) line, were purchased from Horizon Discovery (HD 103‐008, Waterbeach, Cambridge, UK).^22^, ^35^ SW48 cells were cultured in RPMI‐1640 medium containing 10% fetal bovine serum (FBS), 100 units/mL penicillin, 100 mg/mL streptomycin, and 2 mM l‐glutamine. SW48/TP53 cells were cultured in RPMI 1640 medium containing 2 mM l‐glutamine and 25 mM sodium bicarbonate supplemented with 10% FBS and 800 µg/mL geneticin (G418). Human WiDr (missense mutation *TP53* R273H^+/+^) colon cancer, OVCAR‐3 (missense mutation *TP53* R248Q^+/+^) ovarian carcinoma and MCF‐12A noncancerous mammalian epithelial cell lines were purchased from American Type Culture Collection (ATCC; Manassas, VA). Cells of WiDr and OVCAR‐3 lines were cultured in RPMI‐1640 or ATCC‐formulated EMEM containing 10% FBS, 100 units/mL penicillin, 100 µg/mL streptomycin and 584 mg/L l‐glutamine. MCF‐12A cells were cultured in Dulbecco's modified Eagle's medium‐F12 (1:1) supplemented with 5% horse serum, insulin (5 μg/ml), hydrocortisone (500 ng/ml), human epidermal growth factor (20 ng/ml), and cholera toxin (100 ng/ml). Cells were maintained in an incubator humidified with 95% air and 5% CO_2_ at 37 ºC. SW48‐Dox and SW48/TP53‐Dox, which are sublines of SW48 and SW48/TP53 cells, were cultured in 10% FBS RPMI‐1640 medium containing 25 nM doxorubicin (Dox) for 16 weeks (~26 passages).

### 2.2 Cell viability assay

Cell viability was assessed using the CellTiter‐Glo luminescent cell viability assay kit (Promega, Madison, WI), as described previously.^22^, ^23^ Briefly, cells (4000 cells/well; 2500 cells/well for MCF‐12A) were grown in 96‐well plates overnight and then switched to 5% FBS medium containing drugs for 72 hours treatments. For combination treatment, cells were cultured in 5% FBS medium containing respective agents for 48 hours in advance and then cocultured with drugs for an additional 72 hours. Cell viability was assessed in a Synergy HT microplate reader (BioTek, Winnooski, VT, USA), following incubation with CellTiter‐Glo reagent. A new GCS inhibitor, Genz‐161 (GENZ 667161, (*S*)‐quinuclidin‐3‐yl(2‐(2‐(4‐fluorophenyl)thiazol‐4‐yl)propan‐2‐yl carbamate) was kindly provided by Sanofi Genzyme (Framingham, MA).^36^, ^37^ Doxorubicin hydrochloride (Dox) was purchased from Sigma‐Aldrich (St. Louis, MO, USA). Monosialoganglioside (NH_4_^+^ salt, GM2), disialoganglioside (NH_4_^+^ salt; GD3), and globotriaosylceramide (Gb3) were purchased from Matreya (State College, PA, USA).

### 2.3 Induced apoptosis and imaging flow cytometry assay

Induced apoptosis with flow cytometric assessment was carried out using an APO‐DIRECT kit (BD Biosciences, San Jose, CA, USA) and propidium iodide (PI), as described previously.^20^, ^23^ Briefly, cells were pretreated with Genz‐161 (1 μM) or vehicle in 5% FBS medium for 48 hours, and then cotreated with Genz‐161 (1 μM) and Dox (100 nM) for an additional 48 hours to induce apoptosis. After fixing with paraformaldehyde (1% w/v) and 70% ethanol, cells (1 x 10^6^ cells/ml) were incubated with FITC‐labeled Br‐dUTP (5 μg/100 μL; FITC BRDU) and then PI (5 μg/100 μL) in staining buffer containing RNase for apoptosis detection. For cell cycle analysis, fixed cells (1 x 10^6^ cells/ml) were incubated with PI (5 μg/100 μL) in staining buffer containing RNase. Cells were then analyzed using an imaging flow cytometry system, namely an ImageStreamX Mark II with high‐resolution microscope (Amnis, Seattle, WA, USA). For each sample, 5000 events were counted in triplicate. Data were analyzed with the IDEAS^®^ Software package (Amnis), and the gate for apoptotic cells was defined by cells with FITC‐labeled Br‐dUTP (FITC BRDU, green).^20^

### 2.4 Cellular GCS activity and HPLC analysis

Cellular Cer glycosylation and analysis were performed as described previously.^38^, ^39^, ^40^ Briefly, cells (1 × 10^6^ cells/100‐mm petri dish) were grown in 10% FBS RPMI‐1640 medium for 24 hours and then were cultured in 5% FBS RPMI‐1640 medium containing Genz‐161 for an additional 48 hours. For assessing cellular Cer glycosylation, the treated cells (10^6^ cells/reaction) were incubated with 2.0 µM of NBD C_6_‐Cer (complexed to BSA) in 1% BSA RPMI‐1640 medium (200 µl) at 37ºC for 2 hours, with gentle shaking (40 rpm). After rinsing with ice‐cold 10 μM phosphate‐buffered saline (PBS, pH 7.4, three times), cells suspended in ice‐cold acidic methanol (acetic acid: methanol, 1:50, v/v) were transferred into glass vials (1.3 mL), and mixed with chloroform (1.3 mL) and water (1.3 mL) to extract cellular lipids. After separation, the organic lower phase was collected and evaporated to dryness, and the residue was then stored at −20ºC until further analysis.

For HPLC assay, the extracted lipids were dissolved in 100 µL of chloroform/methanol/*ortho*‐phosphoric acid (80:20:0.1, v/v/v; equal fluorescence of ~200 FU in 100 µL for all samples). Each sample (5 μL) was loaded by autosampler injection onto a normal‐phase silica column (5 µm ZORBAX Rx‐SIL, 4.6 mm × 250 mm) and eluted by linear gradient (0‐14 minutes, 1 mL/min) formed with solvent system A (chloroform/methanol/*ortho*‐phosphoric acid) (80:20:0.1, v/v/v) and solvent system B (chloroform/methanol/H_2_O/*ortho*‐phosphoric acid) (60:34:6:0.1, v/v/v/v). NBD sphingolipids were detected by fluorescence using excitation and emission wavelengths of 470 and 530 nm, respectively in an HPLC system (1220 Infinity LC Gradient System VL) equipped with an Agilent 1260 fluorescence detector (Agilent, Santa Clara, CA). The fluorescence peaks for NBD C_6_‐GlcCer and NBD C_6_‐Cer were identified by comparing their retention times with those of standards, and GlcCers were quantitated against standard curves. Each sample was analyzed at least three times, and calculated GCS activities are as normalized against total cellular proteins.

### 2.5 GEM preparation and characterization

GEMs of cells or tissues were prepared and characterized as described previously.^11^, ^15^, ^16^ Briefly, cells (1.2 × 10^7^ cells) were suspended in 2 mL of lysis buffer (1% Triton X‐100 in TNEV solution, 10 mM Tris‐HCl, pH 7.5, 150 mM NaCl, 5 mM EDTA, 1 mM phenylmethylsulfonyl fluoride, 1 mM Na_3_VO_4_), and incubated on ice for 1 hour. Tissues of mice (~120 mg) were finely shredded with scissors and homogenized with a sonicator in the same lysis buffer, and then incubated on ice for 1 hour. Lysates of cells or tissues were centrifuged at 1300 *g* for 5 minutes to remove nuclei and large cellular debris. Samples of supernatant (1.5 mL) were overlaid onto the gradient sucrose solution (2.5 mL each of 80%, 40% and 5% sucrose from bottom to top) in SW41 centrifuge tubes, which were then centrifuged at 100, 000 *g* at 4ºC for 42 hours. Each fraction (800 μL) of gradient solution after ultracentrifugation was collected from the top to bottom (fractions 1‐10). The protein concentrations of these fractions were assessed by using a BCA protein assay kit. Equal protein amounts (12 μg in 20 μL) of each fraction or the fraction 4 of samples were mixed with the loading buffer and subjected to SDS‐PAGE and immunoblotting. Mouse anti‐human flotillin‐2 monoclonal antibody (B‐6) purchased from Santa Cruz Biotechnology (Santa Cruz, CA) was used to recognize flotillin‐2 (Flot2), a GEM component.^11^ Cells were pretreated with Genz‐161 (1 μM, 48 hours) or vehicle, and then cotreated with doxorubicin (100 nM) for an additional 24 hours.

### 2.6 Dot blotting of globotriaosylceramide (Gb3)

Dot blot was performed as described previously, with minor modification.^41^, ^42^ Briefly, nitrocellulose membranes were coated with Shiga toxin 1B (STxB, 0.2 μg/mL in PBS) and dried in air. The fractions collected from different samples were normalized against proteins in the fraction 4 (0.5 μg) with TNE buffer after preparation. Equal volumes (4 μL) of each fraction were spotted onto the precoated nitrocellulose membrane, and completely dried in air. After wash with PBS containing 0.05% Tween‐20 (PBST) and block with 2% BSA in PBST, the membrane strips were incubated with anti‐CD77/Gb3 primary antibody (1:500) for ~12 hours at 4ºC, and an additional 2 hours at room temperature. The immunoblots were further incubated with horseradish peroxidase (HRP)‐conjugated anti‐mouse IgG (1:5000 dilutions), and the images were detected using SuperSignal West Femto substrate (Thermo Fisher Scientific). Mouse anti‐human CD77/Gb3 antibody (cat. 551352, clone 5B5 RUO, IgM) was purchased from BD Bioscience (San Jose, CA). Shiga toxin 1B‐subunit (STxB) was kindly provided by Dr. Anne V. Lane (Division of Geographic Medicine and Infectious Diseases, Tufts Medical Center, and Tufts University School of Medicine, Boston, MA) ^43^.

### 2.7 Immunoprecipitation of lipid‐associated proteins

The requisite immunoprecipitation (IP) assay was performed as described previously, with minor modification.^15^, ^44^ Pierce protein L‐conjugated magnetic beads (2.0 mg/mL, cat.88849; 10 μL) were incubated with anti‐CD77/Gb3 (2 μg) or p53 antibody (2 μg, used as a non‐GEM control, see below) in 500 μL TNE buffer (10 mM Tris‐HCl, pH 7.5, 150 mM NaCl, 5 mM EDTA, 1 mM PMSF) at room temperature for 120 m, shaking at 60 rpm. After washing three times with TNE buffer and magnet, the antibody‐protein L beads were resuspended in 300 μL TNE buffer and mixed with 200 μL GEMs (fraction 4, prepared above). After 14 hours incubation at 4ºC, the GEM‐antibody beads were washed three times and suspended in 30 μL PBST buffer (equal to 75 μg GEM proteins) for further SDS‐PAGE separation and immunoblotting. The GEMs (fraction 4, 4 μL or 10 μg GEM proteins) were directly loaded, as input. Mouse monoclonal p53 antibody (sc‐126, DO‐1) was purchased from Santa Cruz Biotechnology (Dallas, TX).

### 2.8 Western blot analysis

Western blotting was carried out as described previously.^22^, ^23^, ^45^ Briefly, cells or tissue homogenates were lysed in NP40 cell lysis buffer (Biosource, Camarillo, CA) to extract total cellular proteins following these treatments. Equal amounts of proteins (50 µg/lane) were resolved by using 4‐20% gradient SDS‐PAGE (Life Technologies). The nitrocellulose‐membrane blots transferred were blocked in 5% fat‐free milk in 0.05% Tween‐20, 20 mM phosphate‐buffered saline, pH 7.4 (PBST), and then incubated with each one of the primary antibodies (1:500 or 1:5000 dilution), at 4ºC overnight. These blots were incubated with corresponding horseradish peroxidase‐conjugated secondary antibodies (1:5000 dilutions) and detected using SuperSignal West Femto substrate (Thermo Fisher Scientific). Glyceraldehyde‐3‐phosphate dehydrogenase (GAPDH) was used as a loading control for cellular protein. Antibodies against human p53 phosphorylated at Ser15 were purchased from Cell Signaling Technology (Danvers, MA). Antibodies for Puma, p21Waf1/Cip1 (p21), Bax, p53, and GAPDH were obtained from Santa Cruz Biotechnology (Dallas, TX). Relative protein levels were calculated from optical density values for each protein band, normalized against those for GAPDH from three separate blots.

### 2.9 Animal studies in tumor‐bearing mice and transgenic mice

All animal experiments were approved by the Institutional Animal Care and Use Committee, University of Louisiana at Monroe (ULM), and were carried out in strict accordance with good animal practice as defined by NIH guidelines. Athymic nude mice (Foxn1^nu^/Foxn1^+^, 4—5 weeks, female) were purchased from Harlan (Indianapolis, IN) and maintained in the vivarium at ULM. Animal studies were conducted as described previously ^22^, ^23^. Briefly, cell suspension of SW48 or SW48/TP53 (5‐7 passages, 1 × 10^6^ cells in 20 μL/mouse) was subcutaneously injected into the left flank of the mice. Mice with tumors (~2 mm in diameter) were randomly allotted to different groups (five mice/group) for treatments. For treatments, Doxorubicin (Dox, 200 μg/kg once every 6 days) was administrated intraperitoneally alone or with PDMP (4.0 mg/kg once every 3 days) for 32 days. Mice were monitored by measuring tumor sizes, body weights, and clinical observations, twice a week. Tumors and other tissues were dissected and stored at −80ºC for further analyses.

### 2.10 Immunocytochemistry

Immunocytochemistry assessments were accomplished as described previously.^23^, ^45^ Briefly, cells of WiDr or SW48‐Dox lines were pretreated with Genz‐161 or transfected with siRNA‐Gb3 synthase (100 nM) for 48 hours, then placed (8000/chamber) in four‐chamber slides and cotreated with 100 nM doxorubicin for 48 hours in 5% FBS medium. After wash and fixation, cells were blocked with 5% goat serum in PBS for 30 minutes at room temperature, and then incubated with primary antibodies, anti‐p21 (1:200), ‐phosphorylated p53 (pp53), and ‐METTL3 (1:1500) in blocking solution, at 4ºC overnight. Corresponding Alexa Fluor 488‐conjugated anti‐mouse IgG and Alexa Fluor 555‐conjugated anti‐rabbit IgG (1:1000) were applied for further incubation to recognize the corresponding primary antibodies. After washing, cell nuclei were counterstained with DAPI (4̍,6‐diamidino‐2‐phenylindole) in mounting solution (Vector laboratories, Burlingame, CA, USA). Images (200 × magnification) were captured with an Olympus BX63 automated fluorescence microscope with monochrome CCD camera (Olympus, Tokyo, Japan). Alexa Fluor 488‐conjugated anti‐mouse IgG and Alexa Fluor 555‐conjugated anti‐rabbit IgG were purchased from Thermo Fisher Scientific.

### 2.11 Data analysis

All experiments were repeated more than two times. Data are expressed as mean ±SD. Two‐tailed Student's *t* tests and ANOVA were used to compare the continuous variables in groups, using the Prism v7 program (GraphPad Software, La Jolla, CA). All *P* < .05 comparisons were regarded as statistically significant.

## 3 RESULTS

### 3.1 Ceramide glycosylation catalyzed by glucosylceramide synthase correlates with drug resistance in mutant p53‐containing colorectal cancer cells

**Figure 1:**
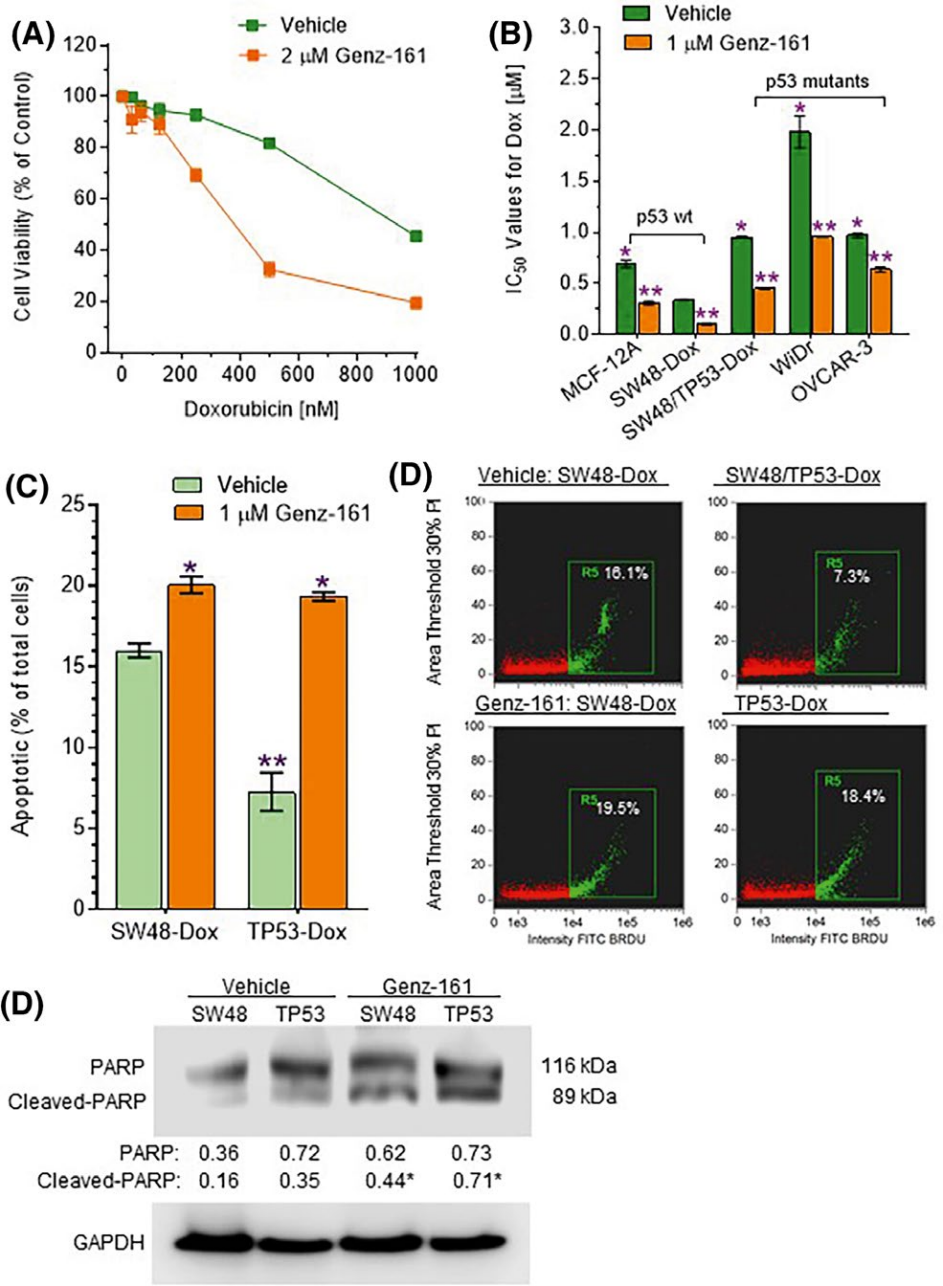
Genz‐161 sensitizes cancer cells carrying *TP53* mutations to doxorubicin cytotoxicity. A, Doxorubicin response in SW48/TP53‐Dox cells pretreated with Genz‐161. Cells were pretreated with 2 μM Genz‐161 or DMSO (vehicle) for 48 hours, and then cotreated with doxorubicin (Dox, 0‐1000 nM) for an additional 72 hours. *, *P* < .01 compared with vehicle control. B, IC_50_ values for Dox treatments of colon cancer cells. Cells were pretreated with 1 μM Genz‐161 or DMSO (vehicle) for 48 hours, and then cotreated with Dox (0‐1000 nM) for an additional 72 hours. *, *P* < .01 compared with *TP53* wild‐type SW48‐Dox cells; **, *P* < .01 compared with vehicle control. C, Dox‐induced apoptosis in cells. Cells of SW48‐Dox or SW48/TP53‐Dox were pretreated with 1 μM Genz‐161 or DMSO (vehicle) for 48 hours, and then cotreated with 100 nM Dox for additional 48 hours to induce apoptosis. Apoptotic cells were identified and analyzed by using ImageStream X Mark II system, and the data analyzed with IDEAS® Software. *, *P* < .01 compared with vehicle control; **, *P* < .01 compared with SW48‐Dox cells. D, Apoptosis assay with imaging flow cytometry. FITC BRDU, bromodeoxyuridine‐FITC. Apoptotic cells labeled with FITC BRDU (green) are presented as percentages of total cells detected in each sample. E, Western blot. Cells were pretreated with 1 μM Genz‐161 or vehicle for 48 hours, and then cotreated with 100 nM Dox for an additional 48 hours to induce apoptosis. Equal amounts of total proteins (50 μg/lane) were resolved and immunoblotted. *, *P* < .01 compared with cells treated with Dox alone

Colon cancer TP53‐Dox cells, which heterozygously carried R273H mutant p53 (*TP53* R273^/+^, introduced by a CRISPR/Cas9 system in SW48 cells) and then were subjected to long‐term subchronic exposure to doxorubicin (Dox), displayed resistance to anticancer drugs.^20^, ^22^ We pretreated TP53‐Dox cells with Genz‐161 (1 μM for 48 hours), a new selective GCS inhibitor,^36^, ^37^ and found that Genz‐161 pretreatments significantly increased sensitivity of cells to Dox (Figure 1A). Compared with SW48‐Dox cells carrying only wild‐type *TP53* alleles, the IC_50_ values for Dox in cells of the *TP53* mutant lines studied (TP53‐Dox (R273H^/+^), WiDr (R273H^+/+^) and OVCAR‐3 (R248Q^+/+^)) were higher by three‐ to sixfold (Figure 1B). Interestingly, pretreatments of these cells with Genz‐161 (1 μM, 48 hours) resensitized p53 mutant cells substantially, decreasing Dox IC_50_ values by approximately twofold in TP53‐Dox, WiDr and OVCAR‐3 cells (Figure 1B). Dox treatments (100 nM for 48 hours) induced apoptosis in SW48‐Dox cells, but the same treatment regimen effected apoptotic death of a substantially smaller fraction of TP53‐Dox cells (Figure 1C,D). In analysis with imaging flow cytometry, we found that Genz‐161 pretreatments substantially increased Dox‐induced apoptosis, by threefold (19.3% vs 7.2% total cells, *P* < .001), in TP‐53‐Dox cells. However, Genz‐161 pretreatments only slightly increased Dox‐induced apoptosis, by approximately 20%, in SW48‐Dox cells (Figure 1C,D). Furthermore, Western blot showed that Genz‐161 pretreatments substantially increased the levels of cleaved PARP, by more than twofold in TP53‐Dox and SW48‐Dox cells treated with Genz‐161 and Dox, as compared with Dox treatment alone (Figure 1E).

**Figure 2:**
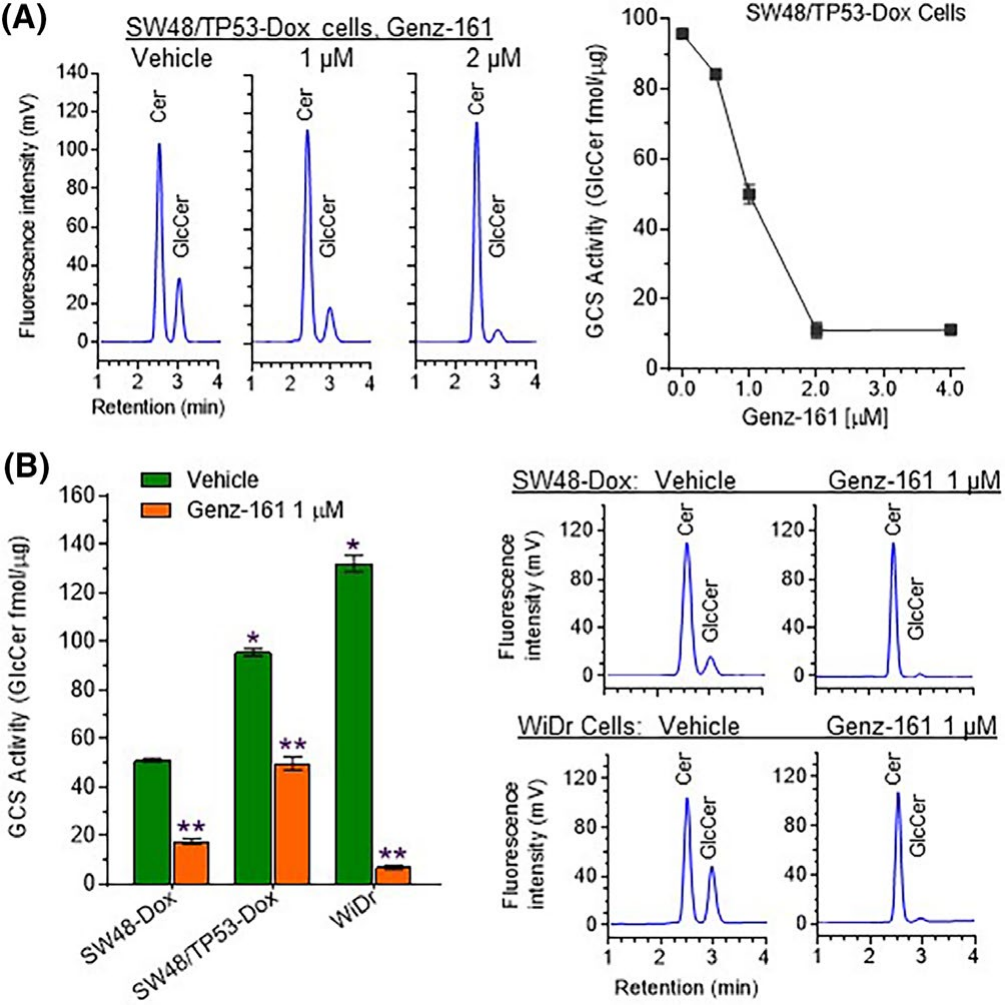
Genz‐161 inhibits the activities of glucosylceramide synthase in cancer cells. After treatments with Genz‐161 (0, 0.5‐4 μM, or 1 μM) for 48 hours, cells were incubated with NBD C_6_‐ceramide (1 μM for 2 hours) in 1% BSA medium. GCS activities are derived from the amounts of NBD‐GlcCer produced (calculated from an NBD‐C_6_‐GlcCer standard curve), normalized vs total cellular protein. A, Representative HPLC chromatograms obtained for TP53‐Dox cells. Cer, NBD‐C_6_‐ceramide; GlcCer, NBD‐C_6_‐glucosylceramide. B, Response versus Genz‐161 concentration: GCS activities in TP53‐Dox cells. C, GCS activities of cancer cells of various lines treated with 1 μM Genz‐161. *, *P* < .01 compared with SW48‐Dox cells treated with vehicle; **, *P* < .01 compared with vehicle of each cell line. D, Representative HPLC chromatograms for SW48‐Dox and WiDr cells. Cer, NBD C_6_‐ceramide; GlcCer, NBD C_6_‐glucosylceramide

Previous reports showed that systemic administration of Genz‐161 significantly reduced GlcCer and other glycosphingolipids in brain tissues of mice (K14) in an animal model for Gaucher disease, and in a parallel cell model (CBE‐N2a).^36^, ^37^ It remained unclear, however, whether or not Genz‐161 would inhibit GCS in human cancer cells or tumors comprised therefrom to any productive end. We therefore assessed the effects of Genz‐161 on GCS activity in colon cancer cells. In TP53‐Dox cells, Genz‐161 treatments (0.5‐4 μM, 48 hours) reduced cellular GlcCer levels in a concentration‐dependent manner (Figure 2A, right). Genz‐161 treatments inhibited GCS activities to approximately 50% at 1 μM, and 90% at 2 μM concentrations in TP53‐Dox cells (Figure 2A left). Compared to drug‐sensitive SW48‐Dox cells, GCS activities were seen to be significantly higher in drug‐resistant cancer cells, by approximately twofold (96 vs 51 fmol/μg, *P* < .001) in TP53‐Dox, and by sevenfold (132 vs 18 fmol/μg, *P* < .001) in WiDr cells (Figure 2B, left). Furthermore, we found that Genz‐161 treatments (1 μM, 48 hours) significantly inhibited GCS activities, to 35% (18 vs 51 fmol/μg, *P* < .001) in SW48‐Dox cells, to 51% (49 vs 96 fmol/μg, *P* < .001) in TP53‐Dox cells, and to 5% (7 vs 132 fmol/μg, *P* < .001) in WiDr cells, respectively (Figure 2B, right). Altogether, these results indicate that enhanced Cer glycosylation catalyzed by GCS protected cancer cells carrying *TP53* mutations in response to Dox. Conversely, Genz‐161 inhibition of GCS/Cer glycosylation resensitized these p53 mutant cells to the genotoxic anticancer drug doxorubicin.

### 3.2 Suppression of ceramide glycosylation modulates Gb3 levels in GEMs of cancer cells

**Figure 3:**
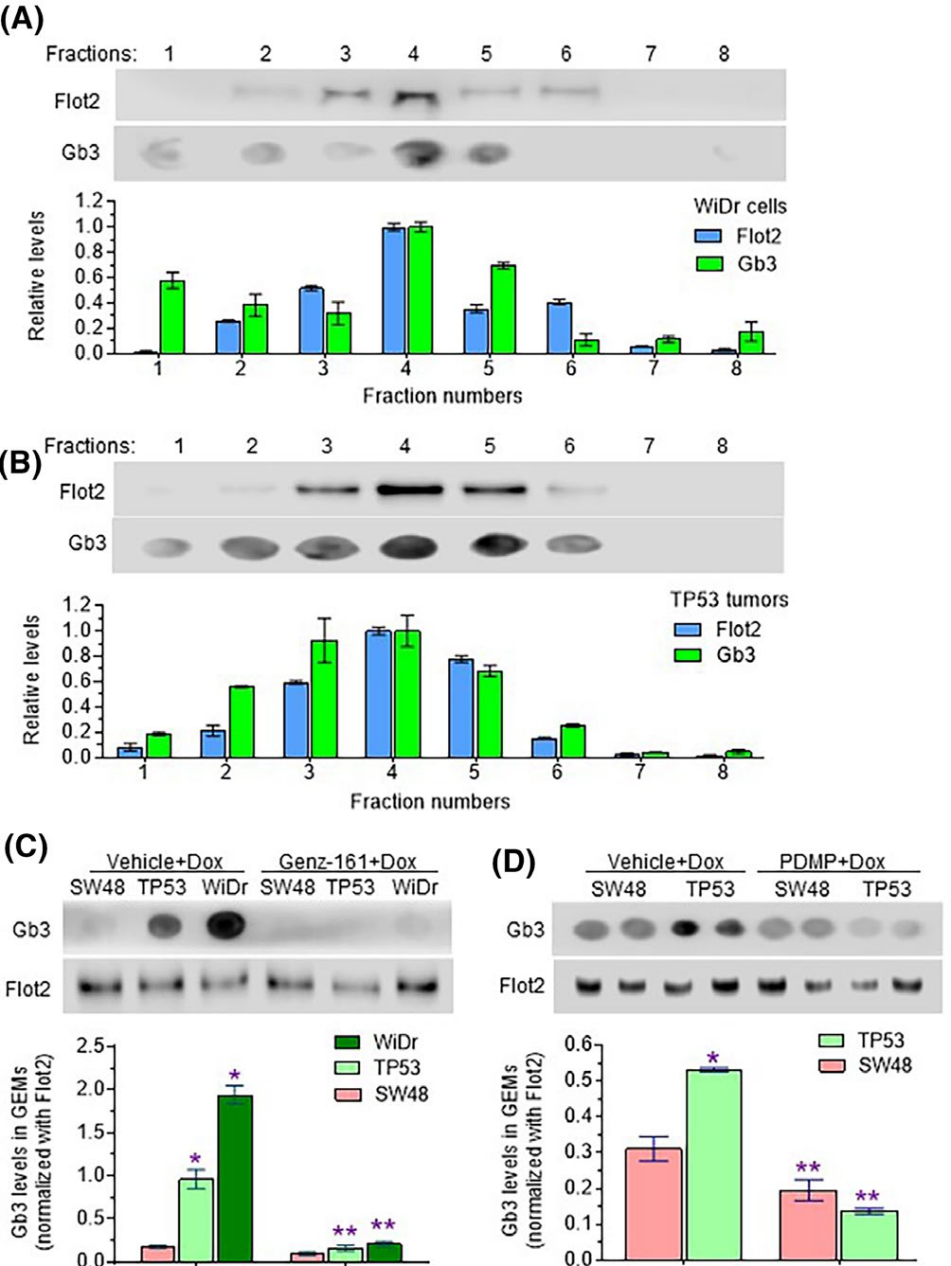
GCS inhibition suppresses Gb3 in GEMs of cancer cells and tumors. A, GEMs‐Flot2 in WiDr cancer cells. After treatments with Dox (100 nM, 3 days) and lipid rafts isolation, equal volumes (4 μL) of fractions from WiDr cancer cells were resolved on SDS‐PAGE for immunoblotting (top image strip) and spotted onto nitrocellulose membrane for dot blot (bottom image strip). B, GEMs‐Flot2 in tumors. Equal volumes (4 μL) of fractions from SW48/TP53 tumors in mice treated with Dox (200 μg/kg, *i*.*p*, once every 6 days for 32 days were resolved on SDS‐PAGE for immunoblotting (top image strip) and spotted onto nitrocellulose membrane for dot blot (bottom image strip). C, Effects of Genz‐161 on Gb3 in GEMs of cancer cells. Cells of SW48‐Dox (SW48) and SW48/TP53‐Dox (TP53) lines were pretreated with 1 μM Genz‐161 or vehicle for 48 hours, and then cotreated with Dox for an additional 48 hours. Equal amounts of total proteins (0.4 μg/lane) of the fraction 4 obtained from cell lysate ultracentrifugation were used for dot blot and immunoblotting. Representative images of dot blot and Western blot are presented in the top and bottom strips, respectively. *, *P* < .01 compared with SW48‐Dox cells treated with Dox; **, *P* < .01 compared with Dox alone of each cell line. D, Effects of PDMP on Gb3 levels in GEMs of tumors. Mice bearing tumors from SW48 and SW48/TP53 (TP53) inoculation were treated with Dox alone (200 μg/kg, *i*.*p*, once every 6 days for 32) or with PDMP (4.0 mg/kg, *i*.*p*, once every 3 days) for 32 days. Equal volumes (4 μL) of fractions were applied for dot blots or Western blots. Representative images of dot blot and Western blot are presented in the top and bottom strips of image, respectively. *, *P* < .01 compared with SW48 tumors of mice treated with Dox alone; **, *P* < .01 compared mice treated with Dox alone

Doxorubicin treatments induced *de novo* ceramide synthesis, and inhibition of GCS further enhanced ceramide levels in cancer cells.^22^, ^32^, ^33^ Our previous studies with inhibition of ceramide synthases indicated that GSLs, not ceramide, were associated with expression of missense p53 R273H mutant.^20^, ^22^ To elucidate how increased glycosphingolipids (GSLs) serves to protect p53 mutant cells from Dox, we firstly assessed GSL‐enriched microdomains (GEMs) in cells and in tissues. GEMs are subdomains of plasma membrane that are insoluble with cold detergents, and contain a pronouncedly higher proportion of GSLs and lower cholesterol content than other subdomains.^46^ Flot‐2 is a marker for GEMs distinct from caveolar lipid rafts, and requisite involvement of this protein in various signaling pathways has been implicated in the progression of cancer and metastasis formation.^11^ After isolation by gradient‐ultracentrifugation following lysis with Triton X‐100 detergent, GEMs, as signified by Flot‐2 Western blotting, were mainly located in fractions 3‐6 from cells, as exemplified with WiDr cells (Figure 3A). Furthermore, dot blotting showed that Gb3 was also mainly located in fractions 2‐5, prominently in fraction 4 from WiDr cells as well as from cells of other lines. In tumors and colons, GEMs‐Flot2 and Gb3 were mainly present in fractions 3‐5, as exemplified with tumors generated from TP53‐Dox cells (Figure 3B).

We examined the effects of inhibition of Cer glycosylation on the levels of Gb3 in GEMs of cancer cells (fraction 4). Exposure to Dox substantially enhanced the relative levels of Gb3 in GEMs of drug‐resistant TP53‐Dox or WiDr cells, as compared to SW48‐Dox cells (Figure 3C). Genz‐161 treatments (1 μM, 48 hours) significantly reduced Gb3 levels in GEMs of TP53‐Dox cells, by fivefold (0.17 vs 0.96, *P* < .001), and by ninefold (0.21 vs 1.94, *P* < .001) in WiDr cell GEMs, respectively (Figure 3C). Furthermore, the Gb3 levels in GEMs from TP53 tumors in mice treated with Dox (200 μg/kg, once every 6 days for 32 days) were 174% (0.54 vs 0.31, *P* < .001) those of wt p53‐homozygous SW48 tumors under the same treatment (Figure 3D). We had previously reported that PDMP treatments (4.0 mg/kg, *i.p*, once every 3 days for 32 days) in combination with Dox significantly decreased tumor growth and tumor weight in TP53 tumor‐bearing mice, compared to Dox‐alone treatments ^22^. Here, we report that PDMP with Dox treatments also dramatically decreased Gb3 levels, by fourfold (0.13 vs 0.54, *P* < .01), in TP53 tumors, but only slightly reduced Gb3 in SW48 tumors (Figure 3D). Previous studies wherein GSLs were quantified by mass spectrometry showed that the levels of globo‐series GSLs, particularly Gb3 (or Gb3Cer/CD77), were significantly increased in patients’ colorectal cancers,^47^, ^48^ and that inhibition of GCS with Genz‐161 or PDMP significantly decreased the levels of Gb3 and other GSLs in cancer cells, as well as in tissues of animals modeling Gaucher disease.^36^, ^49^ Our present results also indicate that levels of the glycosphingolipid Gb3 in membrane GEMs are consistent with Cer glycosylation in cells or tumors. Conversely, suppression of Cer glycosylation in cancerous cells substantially reduces GEM Gb3 levels.

### 3.3 Gb3 interacts with cSrc and mediates cSrc levels in GEMs of cancer cells in response to ceramide glycosylation

**Figure 4:**
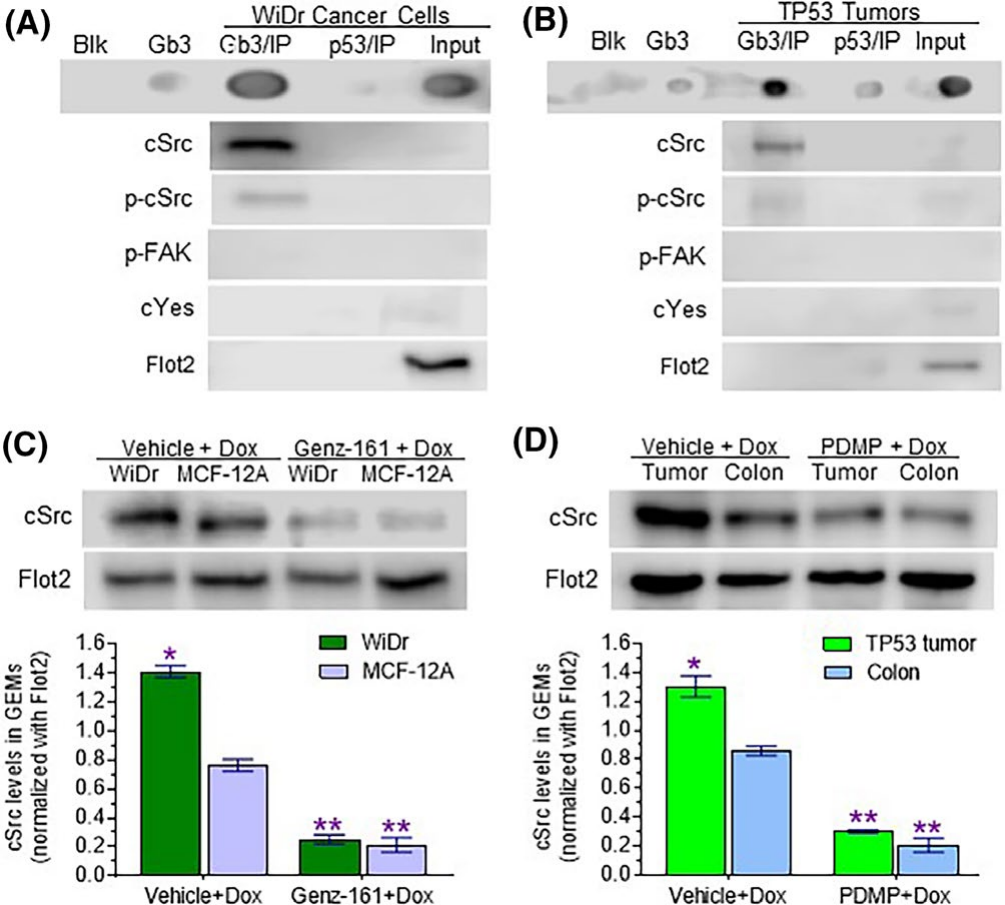
Gb3 is correlated with cSrc in the GEMs of cancer cells and tissues. A, Gb3 immunoprecipitation from GEMs prepared from WiDr cells. Cells were treated with Dox (100 nM, 1 day). Equal volumes (600 μL) of GEMs (fraction 4) after immunoprecipitation with anti‐Gb3 antibody or anti‐p53 antibody (normal IgG) were applied for dot blot and Western blot. B, Gb3 immunoprecipitation from GEMs prepared from tumors. Mice bearing SW48/TP53 tumors were treated with Dox (200 μg/kg, *i*.*p*, once every 6 days for 32 days). Equal volumes (600 μL) of GEMs after immunoprecipitation were applied for Western blot and dot blot. C, Effects of Genz‐161 on cSrc levels in GEMs of cells. Cells of WiDr or MCF‐12A lines were pretreated with 1 μM Genz‐161 or vehicle for 48 hours, and then cotreated with Dox for an additional 48 hours. Equal volumes (4 μL) of GEMs were applied for Western blot. *, *P* < .001 compared with vehicle of each cell line. D, Effects of PDMP on cSrc levels in GEMs of tissues. Mice bearing tumors SW48/TP53 were treated with Dox alone (200 μg/kg, *i*.*p*, once every 6 days for 32 days) or with PDMP (4.0 mg/kg, *i*.*p*, once every 3 days) for 32 days. Equal volumes (4 μL) of GEMs (fraction 4) were applied for Western blot. *, *P* < .001 compared with colon; **, *P* < .001 compared with vehicle

To explore how GSLs interact with other GEM components and modulate cell response to DNA‐damage stress, we applied immunoprecipitation, and further, dot blot and Western blot, to characterize GEM components in GEMs prepared from cells and tissues. In cancer cells, dot blot detected Gb3 as being prominently present in GEMs of WiDr cells treated with Dox (Figure 4A), precipitating with anti‐Gb3 antibody (Gb3 IP), and corroborating by comparing with Gb3 standard (1 ng), a comparison that also provides a sense of the pronounced levels in the GEMs of the Dox‐treated cells. The results clearly indicate that Gb3 could be specifically precipitated, as previously reported.^15^ In Western blot analysis, strikingly higher relative protein levels of cSrc and phosphorylated cSrc (p‐cSrc) were found in GEMs precipitated with Gb3 antibody, as compared the GEMs precipitated with p53 antibody (used as negative control) or overall GEM input (Figure 4A). Also, and consistently, higher levels of Gb3 and cSrc as well as p‐cSrc presented in GEMs precipitated with anti‐Gb3 antibody versus precipitation with negative control antibody (anti‐p53) in samples prepared from TP53 tumors of mice treated with Dox (Figure 4B). Other proteins of the Src kinase family, for example p‐FAK and cYes, were barely detected in GEMs precipitated with anti‐Gb3 antibody (Figure 4A,B). These results further affirm that Gb3‐cSrc association in GEMs. Further, it was found that cSrc levels in GEMs of WiDr cells were higher, by twofold (1.41 vs 0.76, *P* < .001), than in GEMs of noncancerous MCF‐12A cells (Figure 4C). Genz‐161 treatments, which decreased Gb3 in GEMs (Figure 3C), significantly decreased cSrc levels in WiDr cells, by approximately sixfold (0.24 vs 1.41, *P* < .001), and in MCF‐12A cells by threefold (0.22 vs 0.76, *P* < .001) (Figure 4C). In tissues of mice treated with Dox, cSrc levels in GEMs of TP53 tumors were significantly higher than in colon (Figure 4D). Cotreatments with PDMP in addition to Dox, which decreased Gb3 levels in GEMs of TP53 tumors (Figure 3C), significantly decreased cSrc levels, by approximately fourfold (0.30 vs 1.30, *P* < .001) in both tumors and colon (Figure 4D). These results essentially prove that Gb3 not only complexes with cSrc in GEMs, but also governs cSrc levels in GEMs as well as the extent of its phosphorylation leading to further effects.

### 3.4 Effects of Genz‐161 on the protein expression of p53 and p53‐responsive genes in cancer cells carrying p53 R273H mutations

**Figure 5:**
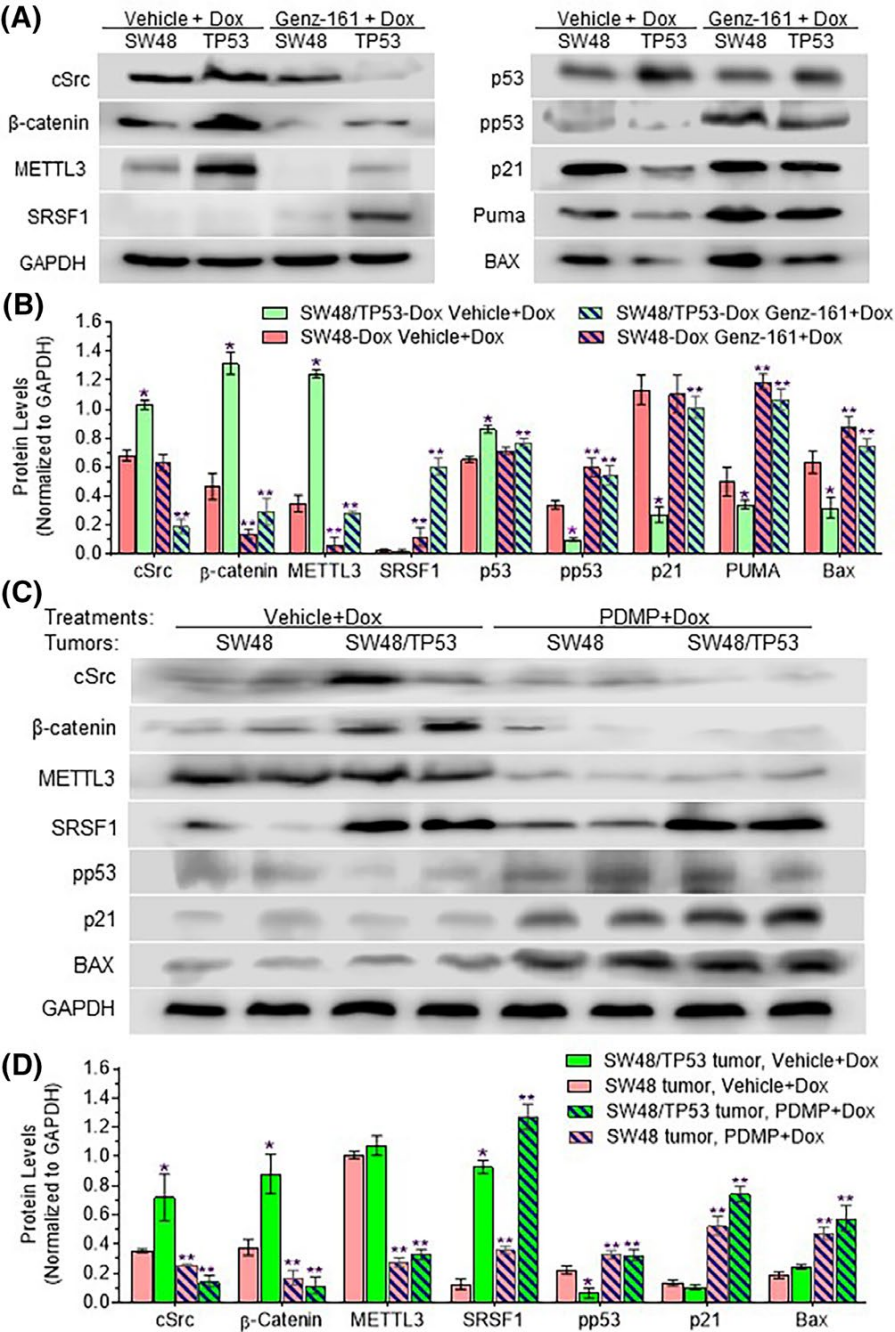
Correlations of the Gb3‐cSrc complex in GEMs with p53 expression of cancer cells and in tumors. A, Representative Western blots of cells treated with Genz‐161 and Dox. SW48‐Dox (SW48) or SW48/TP53‐Dox (TP53) cells were pretreated with Genz‐161 (1 μM) or vehicle for 48 hours, and then cotreated with Dox (100 nM) for an additional 24 hours. Equal amount of proteins (100 μg/lane) were applied for Western blots. B, Relative protein expression levels in cancer cells. *, *P* < .01 compared with SW48‐Dox cells treated with vehicle‐only pretreatment; **, *P* < .01 compared with vehicle‐only pretreatment for the same cell line. C, Representative Western blots of proteins from tumors. Mice bearing xenograft tumors generated from SW48 or SW48/TP53 (TP53) were treated with Dox alone (200 μg/kg, *i*.*p*, once every 6 days) or with PDMP (4.0 mg/kg, *i*.*p*, once every 3 days) for 32 days. D, Relative protein expression levels in tumors. *, *P* < .01 compared with SW48 tumor‐bearing mice treated with Dox alone; **, *P* < .01 compared with Dox‐alone treatments for the same cell line

Previous works showed that alteration of GSLs is correlated to signal transducers in GEMs, and cSrc status and function.^13^, ^14^ To further explore how GSLs modulate protein expression of the p53 R273H mutant, we assessed and characterized the effects of Gb3‐cSrc interaction and β‐catenin on METTL3 expression in SW48/TP53‐Dox colon cancer cells. SW48/TP53 cells treated with Dox expressed significantly higher levels of β‐catenin and METTL3, but lower levels of pp53, p21, and Bax, as compared to SW48‐Dox cells treated with Dox (Figure 5A,B). In combination with Dox, Genz‐161, which inhibited GCS activity (Figure 2B) and decreased Gb3 (and so also cSrc levels) in GEMs (Figure 3C, Figure 4C), significantly decreased levels of β‐catenin, by approximately fivefold (0.28 vs 1.32, *P* < .001), and of METTL3, by fourfold (0.28 vs 1.25, *P* < .001), but increased the levels of SRSF1 by approximately 30‐fold (0.02 vs 0.60, *P* < .01) in SW48/TP53‐Dox cells, as compared to similarly treating these cells but with Dox alone (Figure 5A,B). With these effects, Genz‐161 restored wt p53, increasing levels of pp53 by eightfold (0.08 vs 0.62, *P* < .001), and then protein levels of p53‐responsive genes, including p21, by approximately fourfold (0.27 vs 1.01, *P* < .001) and Bax by twofold (0.35 vs 0.78, *P* < .001) in SW48/TP53‐Dox cells, as compared to treatment with Dox alone (Figure 5A,B). Furthermore, SW48/TP53 tumors of mice treated with Dox expressed significantly higher levels of β‐catenin and METTL3, but lower levels of pp53, p21, and Bax, as compared to SW48 tumors (Figure 5C,D). When combined with Dox treatments, PDMP, which inhibited GCS activity ^22^ and decreased Gb3 (and cSrc) in GEMs of SW48/TP53 tumors (Figure 3D, Figure 4D), significantly decreased the levels of β‐catenin, by approximately sevenfold (0.12 vs 0.88, *P* < .001), of METTL3 by threefold (0.32 vs 1.07, *P* < .001), but substantially increased the levels of SRSF1 in SW48/TP53 tumors and SW48 tumors, compared to Dox treatments alone (Figure 5C,D). With these effects, PDMP restored pp53 levels, increasing them by fivefold (0.32 vs 0.06, *P* < .001), and thence protein levels of p53‐responsive genes, including p21 by approximately sevenfold (0.74 vs 0.11, *P* < .001) and Bax by twofold (0.58 vs 0.25, *P* < .01) in SW48/TP53 tumors, as compared with Dox treatment alone (Figure 5C,D). However, combination treatments of Genz‐161 with Dox only more moderately altered levels of these proteins in SW48 tumors borne by mice, again comparing to Dox treatment alone. These results above indicate that in response to drug treatment, increased Cer glycosylation transactivates the expression of METTL3 by the cSrc and β‐catenin signaling pathway and the resultant m^6^A at the mutant‐codon lends to preferential pre‐mRNA splicing so as to amplify mutant protein production in cancer cells carrying *TP53* R373H mutation. Perhaps of greater interest, aberrant Cer glycosylation was seen to promote m^6^A‐favored pre‐mRNA splicing so as to amplify p53 mutant protein production in cancer cells carrying *TP53* R273H mutation. Suppression of Cer glycosylation with a GCS inhibitor restores wt p53 protein and its function, even in cancer cells carrying homozygous *TP53* R273H mutation.

### 3.5 Effects of Gb3‐cSrc interaction on cell resistance to doxorubicin

**Figure 6:**
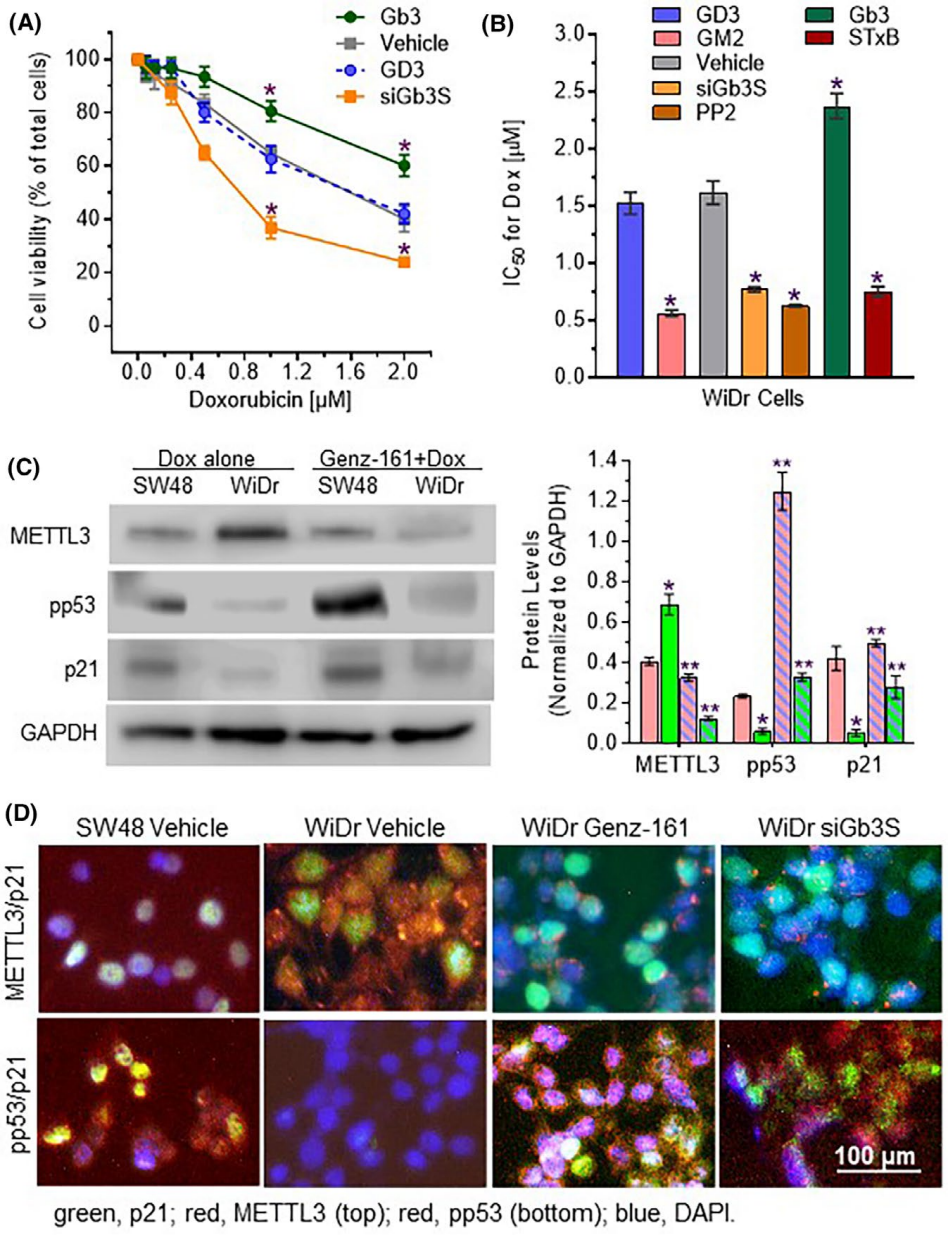
Correlations of the Gb3‐cSrc complex with cell response to doxorubicin in WiDr cells. Cells were pretreated with GM2 (2 μM), GD3 (2 μM), Gb3 (2 μM), siRNA against Gb3 synthase (siGb3S, 100 nM), Shiga toxin B (STxB, 100 nM) and PP2 (100 nM, cSrc inhibitor) or vehicle for 48 hours, and then cotreated with increasing concentrations of Dox for an additional 72 hours. A, Cell responses to treatments. *, *P* < .01 compared with vehicle. B, IC_50_ values for Dox. *, *P* < .001 compared with vehicle. C, Western blotting of cells treated with Genz‐161. Cells of SW48 and WiDr lines were pretreated with Genz‐161 (1 μM) or vehicle for 48 hours, and then cotreated with Dox (100 nM) for 24 hours. Equal amount of proteins (100 μg/lane) were applied for Western blots. *, *P* < .01 compared with SW48 cells treated with vehicle‐only pretreatment; **, *P* < .01 compared with vehicle‐only pretreatment for the same cell line. D, Immunofluorescence of WiDr cells. WiDr cells were pretreated with Genz‐161 (1 μM) or siGb3S (100 nM) for 48 hours and then cotreated with Dox (100 nM) for 24 hours. SW48 Vehicle, SW48‐Dox cells were pretreated with vehicle alone, and then Dox (200 nM) for 24 hours. Green, Alexa Fluor 488−p21; red, Alexa Fluor 555−pp53 (top) or Alexa Fluor 555−METTL3 (bottom); blue, DAPI nucler counterstain

**Figure 7:**
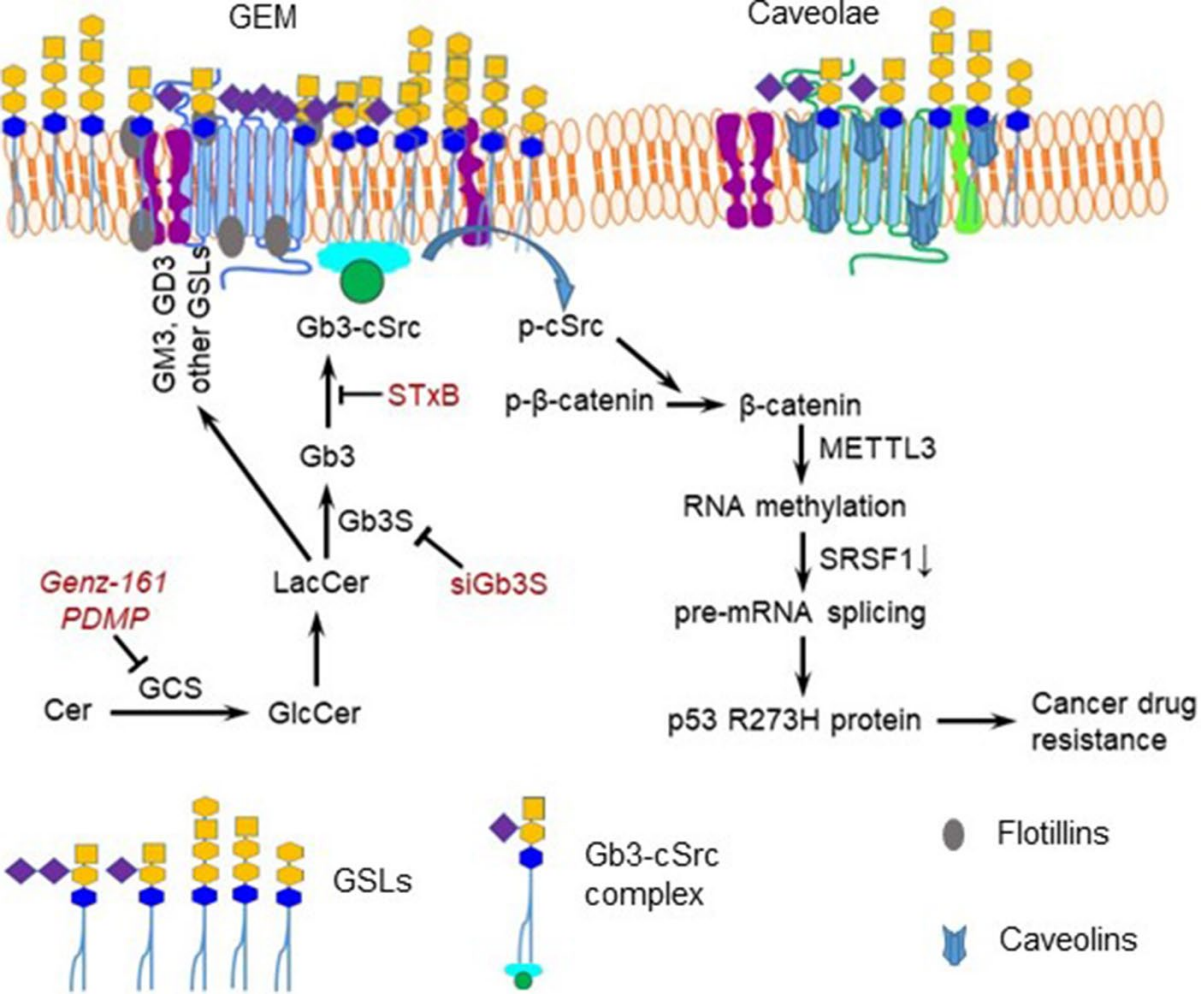
Model for the Gb3‐cSrc correlated mutant p53 signaling pathways. Increased ceramide (Cer) glycosylation by GCS in cancer cells exposed to anticancer drugs in turn increases Gb3‐cSrc association in GEMs and downstream expression of missense p53 mutant protein via the cSrc/β‐catenin signaling pathway and deleterious alteration of pre‐mRNA splicing, thus promoting cancer drug resistance. Conversely, inhibition of GCS with PDMP or Genz‐161, silencing Gb3 synthase with siGb3S, or impeding Gb3‐cSrc interaction with STxB upregulates wild‐type p53 expression and sensitizes cancer cells to anticancer drugs. Gb3 is associated with cSrc in GEMs, possibly owing in part to palmitoylation of Src proteins^8^

We further characterized the impacts of Gb3‐cSrc association on drug resistance in WiDr cells carrying the R273H *TP53* mutation. Previous reports from other laboratories showed that addition of exogenous GM3, Gb3 and GD3 altered cell viability and other behaviors.^50^, ^51^ Shiga toxin 1B subunit (STxB or verotoxin) specifically bound to Gb3 and induced cancer cell death.^52^, ^53^, ^54^ In accord with the above‐described results, Gb3 pretreatment (2 μM), which provides exogenous Gb3 to interact with cSrc, in turn increasing mutant p53 expression, was seen to significantly increase cell viability under Dox treatments (Figure 6A), raising Dox IC_50_ values by 50% (2.4 vs 1.6 μM, *P* < .001) as compared to vehicle pretreatment (Figure 6B). Conversely, siGb3S pretreatments (100 nM), which can silence the expression of Gb3 synthase (Gb3/CD77 synthase or α1,4‐galactosyltransferase, α1,4‐Gal‐T) to decrease Gb3 production, restoring wt p53 in cells ^16^, ^20^, significantly decreased cell viability (Figure 6A), and also the Dox IC_50_ value (by twofold: 0.7 vs 1.6 μM, *P* < .001) (Figure 6B). GD3 is a disialoganglioside GSL that, like Gb3, is generated after Cer glycosylation and enriched in GEMs. However, providing exogenous GD3 by pretreatments (2 μM), unlike with Gb3 pretreatments, did not compromise cell susceptibility to Dox (Figure 6A,B). Interestingly, exogenous GM2 (2 μM) was found to decrease the IC_50_ values for Dox, by approximately fourfold (0.42 vs 1.98 μM, *P* < .001) in WiDr cells. STxB can bind to the gal(α1—4)gal disaccharide moiety of Gb3, and sensitize cancer cells to anticancer drugs or induce apoptosis.^53^, ^54^, ^55^, ^56^ In the present study, STxB (100 nM) resensitized WiDr cells to Dox, significantly decreasing the Dox IC_50_ values, by approximately twofold (Figure 6B). Furthermore, inhibition of cSrc kinase with PP2 (a selective Src kinase inhibitor) also significantly decreased the Dox IC_50_ values, by more than twofold, in WiDr cells (Figure 6B). Parallel effects of Gb3‐cSrc interaction on p53 protein expression in cells are also observed in these cells. As compared to SW48 cells, WiDr cells expressed higher levels of METTL3 and lower levels of pp53 as well as p21 in response to Dox (Figure 6C,D); however, treatments with either Genz‐161 (Figure 6C,D) or siGb3S (Figure 6D) suppressed METTL3, and then increased pp53 as well as p21 in WiDr cells (Figure 6C,D). Altogether, as depicted in Figure 7, these lines of evidence indicate that the Gb3‐cSrc interaction may very well play a key role in regulating the expression of p53‐R273H mutant protein, which could account for cancer resistance to various chemotherapeutic drugs. These results also point to Gb3, rather than other similar GSLs, as being the particular elaborated GSL product of GlcCer that interacts with cSrc kinase and thereby directs cSrc‐signaling pathway forward towards preferential p53 mutant expression and consequent cancer drug resistance.

## 4 DISCUSSION

The present study elucidated that elevated Cer glycosylation leads through crosstalk to increased expression of p53 mutant protein for two different TP53 missense mutations, thus endowing cancer cells with pronounced drug resistance. Anticancer drugs, including doxorubicin and cisplatin, can promote increased Cer glycosylation by upregulating GCS expression in cultured cancer cells and tumors.^32^, ^57^ Extended exposure to lower doses of these drugs can induce drug resistance, as exemplified by cells of the TP53‐Dox line, produced by subchronic Dox treatment of cancer cells (SW48/TP53 *TP53* R273H/^+^) heterozygous for the corresponding *TP53* missense point mutation^20^, ^22^ (Figure 1B). Furthermore, homozygous *TP53* mutation‐carrying cancer cells, either WiDr (*TP53* R273H^+/+^) or OVCAR3 (*TP53* R248Q^+/+^), exhibited greater resistance (Figure 1B).^31^ When comparing GCS activities among cell lines under doxorubicin treatments, we found substantially higher GCS activities in p53 mutant‐carrying cancer cells, particularly in homozygous WiDr cells (Figure 2B). Interestingly, WiDr cells were found to be more sensitive to inhibition of GCS by Genz‐161 than were cells of the TP53‐Dox or SW48‐Dox lines (Figure 2B). Moreover, Genz‐161 significantly resensitized these *TP53* missense mutation‐homozygous cells to the effects of Dox (Figure 1B). These results suggest that the presence of p53 missense mutants may upregulate (or de‐repress) GCS expression to increase GCS activity in these cells. Homozygosity or heterozygosity with respect to *TP53* mutations correlates to innate or acquired drug resistance, respectively, and suppression of ceramide glycosylation with a GCS inhibitor can efficaciously sensitize these cells to anticancer drugs. With LC‐MS analysis, previous studies by others showed that Genz‐161 directly inhibited GlcCer synthesis, and decreased other GSLs in mouse models of neuropathic Gaucher disease and polycystic kidney disease (ganglioside GM3 in the latter).^36^, ^37^, ^58^ Here, we assessed GCS activities in Genz‐161 treated cancer cells and found that Genz‐161 at 1 μM concentration effectively inhibited GCS activity and Gb3 production in all cells tested; Gb3 levels were extensively suppressed even in WiDr cells that exhibited quite high relative levels of GCS activity (132 fmol/μg). Clearly, Genz‐161, a GCS inhibitor, can efficaciously reverse cancer drug resistance of the type being studied here.

Based on our present study, Gb3‐cSrc complex in GEMs evidently plays a key role in increasing the deleterious impacts of cancer drug resistance driven by p53 missense mutant protein. Previous works showed that suppression of GCS restored wild‐type p53 protein expression and its anticancer effects in NCI/ADR‐RES cancer cells carrying a *TP53* deletion‐mutation,^23^, ^45^ and in SW48/TP53 cells heterozygous for a missense *TP53* mutation (R273H^/+^) inserted with CRISPR/Cas9 gene editing.^20^, ^22^ Furthermore, results from our current study indicate that inhibition of GCS with Genz‐161 similarly restored p53 tumor suppression function in WiDr cells homozygous for the *TP53* R273H mutation as well (cf. Figure 6). This surprising finding pushes forward on exploring how GSLs relay elevated Cer glycosylation to favor expression of missense p53 mutant proteins. We identified a linchpin role for the association of Gb3 with cSrc kinase in GEMs of cancer cells in culture (WiDr) as well as in tumors (SW48/TP53 xenograft) (cf. Figure 4A, 4B). Further credence for the importance of Gb3‐cSrc complex emerges from findings that although suppressing Cer glycosylation decreased cSrc levels in cell lines tested (Figure 4), Genz‐161 substantially decreased Gb3 presence in GEMs only in mutant‐p53‐carrying cultured cells and tumors (Figure 3C,D). Furthermore, in a head‐to‐head comparison of isogeneic SW48 (wt‐p53) and SW48/TP53 (mutant‐heterozygous) cells or xenograft tumors generated therefrom, cSrc was only suppressed pronouncedly by GCS inhibition (Genz‐161 or PDMP) in p53‐mutant‐carrying cells, with concomitant pronounced increases of pp53 levels and of most downstream effectors thereof (including Bax with either GCS inhibitor) (Figure 5).

In addition to upregulated expression of GCS in cells under stress, hereditary deficiency of glucosylceramidase (also known as glucocerebrosidase) results in accumulation of GlcCer in cells and causes Gaucher's disease.^59^, ^60^ In Fabry disease, one of the lysosomal storage diseases, *GLA* mutations defect α‐galactosidase A (α‐GALA) in hydrolyzing the terminal α‐galactosyl moieties from GSLs, and bring about accumulation of Gb3 in cells, particularly in kidneys, heart and skin.^61^, ^62^ Aberrant elevation of these GSLs in GEMs can alter membrane fluidity, membrane protein trafficking and assembly of signaling molecules.^8^, ^12^ Understanding how particular GSLs interact with signaling molecules and mediate or modulate cellular signaling pathways is also important for these disorders. Regarding association of GSLs with Src family kinases, LacCer was also found to interact with Lyn (a Src family kinase) and G_αi_ in LacCer‐enriched lipid rafts of neutrophil‐like HL‐60 cells to mediate superoxide generation and phagocytosis.^63^ Associations of gangliosides GM3 and GM2 with TRAF6 and cSrc were found to be required for the receptor clustering of RANK and IGF1R, and subsequent activation of downstream signaling, including MAPK‐dependent pathways for osteoblast differentiation and maturation.^64^ Coexistence of GD3 with Yes in GEMs of SK‐MEL‐28 melanoma cells was found to be involved in the increased cell proliferation and invasion.^65^ In the other hands, cellular ceramides can recruit and activate protein phosphatase 2A, and then either enhance AKT phosphorylation and mTOR signaling for autophagy^66^ or enhance SRSF1 phosphorylation involved in alternative RNA splicing.^67^

In our prior studies, we uncovered that cellular signaling of cSrc and β‐catenin in regulating expression of MDR1, FGF2 and METTL3 involve Gb3.^16^, ^17^, ^20^ Given these results, we applied Gb3 immunoprecipitation and GEM characterization to show that Gb3 interacts with cSrc or phosphorylated cSrc, and not with Yes or phosphorylated FAK, in GEMs of WiDr cells and xenograft tumors generated from cells of SW48/TP53 cell line (once again cf. Figure 4A,B). Furthermore, the observation that exogenously supplied GM2, STxB, or siGb3S increased sensitivity of WiDr cells to Dox comparably to what was seen upon treating cells with the cSrc‐selective kinase inhibitor PP2 provides indirect affirmation of the integral role of the Gb3‐cSrc interaction in pro‐cancerous pathways. More importantly, this study for the first time indicates that Gb3‐cSrc complex in GEMs elevates the expression of missense mutant p53 R273H not only in mutation‐heterozygous cancer cells, but also in mutation‐homozygous ones (WiDr cells). In corroboration with our recent studies,^20^, ^22^ these effects likely involve deleterious mRNA methylation (*N^6^*‐methyladenosine) and consequent altered pre‐mRNA splicing, as indicated by correlations seen here with increased METTL3 and decreased SRSF1 expression levels. Accordingly and conversely, our findings indicate that suppression of Cer glycosylation with a GCS inhibitor, or directly impeding Gb3‐cSrc elevation or interaction, can restore wild‐type p53 function and resensitize drug‐resistant cancerous cells and tumors to DNA‐damage stressing chemotherapeutic agents.

## CONFLICT OF INTEREST

Y. Y. Liu is a member of Scientific Advisory Board for Sanofi‐Genzyme.

## AUTHORS’ CONTRIBUTIONS

Y. Y. Liu, K. R. Roy, R. Hill, and H. Lu designed the research; K. R. Roy, Y. Y. Liu, and S. C. Roy analyzed the data; K. R. Roy, Y. Y. Liu, M. B. Uddin, and S. C. Roy performed the research; Y. Y. Liu, K. R. Roy, R. Hill, Y. T. Li, M. B. Uddin, H. Lu, Y. T. Li, and J. C. Chamcheu wrote the paper; Y. T. Li and J. Marshall contributed new reagents.
